# Transendothelial electrical resistance measurement by a microfluidic device for functional study of endothelial barriers in inflammatory bowel disease

**DOI:** 10.3389/fbioe.2023.1236610

**Published:** 2023-07-14

**Authors:** Ya Li, Min Xu, Zhu Zhu, Feng Xu, Bing Chen

**Affiliations:** ^1^ Department of Gastroenterology, The First Affiliated Hospital of Zhengzhou University, Zhengzhou, China; ^2^ Department of Biological Sample Bank, The First Affiliated Hospital of Zhengzhou University, Zhengzhou, China

**Keywords:** transendothelial electrical resistance (TEER), endothelial cellular barrier, microfluidic device, inflammatory bowel disease (IBD), circulating exosomes

## Abstract

**Introduction:** Inflammatory bowel disease (IBD) is a chronic relapsing and remitting disease with a rising incidence globally. Circulating exosomes play great roles in IBD pathogenesis through exosomal cargoes, especially impacting the function of endothelial barriers. Transendothelial electrical resistance (TEER) measurement is a widely used non-invasive and label-free strategy to monitor endothelial barrier function *in vitro*. This study established a well-designed microfluidic device to monitor the TEER changes of endothelial cellular barrier on-chip after treated with exosome derived from IBD serum.

**Methods:** The chip comprised two layers of microfluidic chambers with top layer for the perfusion of medium to maintain the nutrition and pressure during cell culture, and bottom layer for the extracellular matrix mimic using hydrogel, which are separated by a semipermeable membrane that permitted the formation of endothelial cell barrier. Four electrodes independent from the outlets were integrated to the chip for TEER detection. *In vivo* mouse models mouse models and proteome profiling were performed to finding relevant regulators.

**Results:** With this platform, significant decrease of TEER was detected, indicating that IBD serum exosome impact the endothelial cellular barrier on-chip. *In vivo* mouse models, IBD serum exosome treated group showed great higher DAI scores, shorter colons, more severe histological features, and higher levers of S100A8 expression, promoting the disease progress. Proteome profiling showed that TFRC and ANXA5 have great potentials as novel regulators in IBD.

**Discussion:** This in-house customized microfluidic chip emulates the endothelial barrier microenvironment and enables the TEER monitoring, and can be used to investigate endothelial barrier function *in vitro*. IBD serum exosome promote the severity of disease.

## 1 Introduction

Inflammatory bowel disease (IBD) is a chronic relapsing and remitting disease, including ulcerative colitis (UC) and Crohn’s disease (CD), with a rising incidence globally (Kaplan, 2015; Kaplan and Ng, 2017). UC is characterized by relapsing and remitting mucosal inflammation, initiating in the rectum, and extending continuously to proximal segments of the colon. The most common symptom is bloody diarrhea, the condition diagnosed by colonoscopy and histological examination (Ungaro et al., 2017). In CD, all segments of the gastrointestinal tract can be affected, most commonly the terminal ileum and colon (Torres et al., 2017). Multiple factors, including genetic predisposition, environmental factors, epithelial barrier defects, and mucosal immune dysregulation, contribute to IBD pathogenesis (Xavier and Podolsky, 2007; Eisenstein, 2018). However, its exact pathogenesis is currently still unknown. New findings to delineate the etiology of IBD are necessary for the development of new treatments and, ultimately, achieving disease cure.

The intestinal vascular endothelium is an important barrier in the intestine by regulating the movement of fluid, solutes, and proteins across the endothelium, including blood supply, nutrient transport, tissue fluid homeostasis and immune cell migration, and blocking bacteria penetration (Cromer et al., 2011; Spadoni et al., 2015). Under normal circumstances, no gap exists between endothelial cells, and permeability and leakage are stable and low. In acute inflammation, focal endothelial gaps form temporarily; as a result, the permeability rapidly increases and leakage occurs. In chronic inflammation, blood vessels undergo structural remodeling, manifested as dilation, proliferation (angiogenesis), increased sensitivity to mediators, and continuous gap formation and leakage (Claesson-Welsh et al., 2021). The loss of endothelial barrier function and the resulting activation of angiogenesis, enhancement of vascular permeability, and increase in vascular density and pathological tissue edema promote the recruitment of white blood cells, leading to inflammation and IBD progression (Alkim et al., 2015; Claesson-Welsh et al., 2021). Exosomes are a subset of extracellular vesicles with diameters ranging from 40 to 160 nm; they are made by most cell types and can be detected in almost all biological fluids (Kalluri and LeBleu, 2020; Jeppesen et al., 2023). Increasing numbers of studies indicate that circulating exosomes have great potential in the diagnosis and treatment of IBD and can induce IBD pathogenesis (Zhang et al., 2019; Larabi et al., 2020; Ocansey et al., 2020; Shao et al., 2021). However, the regulatory functions of exosomes on endothelial barriers in IBD are not clear.

Measurement of transendothelial or transepithelial electrical resistance (TEER) is a widely used non-invasive and label-free strategy to monitor the tightness of the endothelial barrier *in vitro*. Currently, both conventional Transwell culture systems and organs-on-chips are used to mimic the endothelial barrier to detect TEER using specific electrodes (Odijk et al., 2015; Srinivasan et al., 2015). As one advantage of microfluidic technologies, organs-on-chips provide a controlled environment to culture cellular barriers, which are more relevant to the *in vivo* microenvironment than traditional *in vitro* culture systems (van der Meer and van den Berg, 2012; Bhatia and Ingber, 2014). Generally, TEER detection chips comprise two layers of microfluidic chambers that are separated by a porous membrane for cellular barrier formation (Douville et al., 2010; Ferrell et al., 2010; Maoz et al., 2017; Wei et al., 2023). To measure the TEER on chip, the integration of electrodes on either side of the porous membrane has also been discussed. For example, to avoid the loss of visual inspection of the cells due to electrodes close to the cellular barrier and improve the reliability and stability, Marinke et al. proposed a direct on-chip TEER measurement method using four electrodes inserted into the two channels without the need to be close to the cells (van der Helm et al., 2016). Ben et al. integrated multi-electrode arrays and TEER measurement electrodes to achieve a dual channel, endothelialized, heart-on-a-chip device for the simultaneous detection of cellular electrical activity and tissue barrier function (Maoz et al., 2017). These engineered systems facilitated the feasible and convenient integration of electrodes for TEER detection.

In this study, we used a well-designed microfluidic chip integrated with four electrodes independent from the outlets to detect the function of the endothelial cellular barrier after treatment with exosomes derived from the serum of patients with IBD. The decrease in TEER indicated that these exosomes significantly impacted the endothelial cell barrier. Moreover, data from the mouse models supported that IBD serum exosomes promote the severity of disease *in vivo*. Proteome profiling was also performed to analyze the exosomal protein cargoes and explore the novel regulators.

## 2 Materials and methods

### 2.1 Device design and fabrication

The two-layer (upper and bottom) microfluidic chip used in this study was designed using LayoutEditor on a chrome mask. The sizes and shapes of the chip on the two layers were identical, and one was bonded to the opposite one with a polyester, porous, semipermeable membrane (Corning) in between. The master mold (silicon wafer) was created by the standard photolithography process. The polydimethylsiloxane (PDMS, Dow Corning) elastomer was fabricated by replica molding from the SU-8 (SU8-2010, MicroChem)/silicon master based on a ratio of 10:1 with the curing agent. After pouring PDMS on the mold, the mixture was degassed in a vacuum chamber for air bubble removal and cured at 80°C for 1 h in the oven. The microfluidic chip was cut out by razor blade, punched for the fluidic connection ports, and then bonded to a glass slide (24 mm × 60 mm) by oxygen plasma. Ag/AgCl electrodes were produced by immersing platinum wire and silver wire into potassium chloride electrolyte and inserted into the designed electrode side of the chip.

### 2.2 Clinical sample collections

Patients with IBD and healthy control individuals were enrolled from the First Affiliated Hospital of Zhengzhou University from December 2021 to March 2022. The inclusion criteria were patients with moderate to severe active IBD who met the diagnostic criteria for IBD. The exclusion criteria were patients with other intestinal organic diseases, other infectious diseases, and pregnant and lactating women. This study was approved by the Ethics Committee of the First Affiliated Hospital of Zhengzhou University (approval number: L2018-Y150), and the patients signed an informed consent form. We collected 5 mL of peripheral blood from each participant into individual serum collection tubes. Serum samples were obtained by centrifuging at 4°C and 3000 rpm for 10 min after coagulation.

### 2.3 Exosome extraction and identification

Serum exosomes were extracted using an exosome extraction kit (KGPE001-10, KeyGEN BioTECH), in which 250 μL of extracellular vesicle separation reagent was added to the serum, gently mixed well, and allowed to stand for 2 h at 4°C. Next, the samples were centrifuged 12,000×g at 4°C for 20 min. The supernatant was discarded to obtain the extracted exosomes, which were stored at −80°C until use. The exosomes were identified through particle size analysis using a nanoparticle tracking analyzer (NTA, ZetaView^®^, Particle Metrix) and image observation using transmission electron microscopy (TEM, HT7700, HITACHI). Biomarkers for exosomes including TSG101, CD63, and Alix proteins were also detected by Western blot.

### 2.4 Cell line and culture conditions

Human umbilical vein endothelial cells (HUVECs) were ordered from Procell Life Sciences Co., Ltd. (Wuhan, Hubei, China) and were cultured in Ham’s F-12K medium complemented with 0.1 mg/mL heparin, 0.03–0.05 mg/mL ECGs, and 10% FBS at 37°C in a humidified atmosphere with 5% (v/v) CO_2_.

### 2.5 TEER measurement

TEER was measured using a lock-in amplifier (HF2LI, Zurich Instruments, Switzerland) operated by a customized LabVIEW program. Before each measurement, the medium inside the chip was refreshed to reach room temperature to minimize measurement errors caused by the changes in temperature and medium conductivity. In the microfluidic chip, two electrodes were located above and below the PC membrane, respectively. The impedance values between any two electrodes were recorded with an AC signal of ∼0.8 V applied to one terminal and the other terminal grounded. The response current was amplified by a current amplifier (HF2TA, Zurich Instruments, Switzerland) with an amplification of 10^8^. Afterward, the amplified signals were fed to the HF2LI for demodulation, with the oscillator frequency set to 10 kHz and the signal sampling rate set to 7K Sa/s. Based on the equivalent resistive circuit and the Gaussian elimination, the TEER value was calculated using Eq. 1, discussed in Section 3.1.

### 2.6 IBD mouse model

An IBD mouse model was induced by DSS (Mw 36,000–50000) treatment. Male C57BL/6 J mice (6–8 weeks, 20–25 g) were ordered from the Experimental Animal Center of Zhengzhou University (Experimental Animal Use License No. SCXK (Yu) 2020 0008) and housed in a specific pathogen-free (SPF) environment at 22°C–26°C and a relative humidity of 40%–70%. All the experiments were performed in strict accordance with good animal practice. The experimental mice were randomly assigned to four groups (n = 6 per group): Control, a negative control group; IBD model, DSS-induced colitis group; IBD model + HC-exos, DSS-induced colitis group treated with exosomes derived from human control; and IBD model + IBD-exos, DSS-induced colitis group treated with exosomes derived from patients with IBD. Mice in IBD model drank 2% DSS water, and those in the control group drank normal water freely. On days 3, 5, and 7, mice in IBD model + HC-exos and IBD model + IBD-exos groups were injected through the tail vein with total protein from 1 mg of exosomes derived from human controls and from patients with IBD. Daily diarrhea, bloody stools, and weight were monitored to calculate the Disease Activity Index (DAI) scores. The experimental mice were euthanized when they showed significant weight loss, with symptoms of curling up, erect hair, slow movement, decreased appetite, and loose stools. The lengths of the colons of these mice were measured. Hematoxylin–eosin (HE) staining was used to identify the severity of the disease, and immunohistochemistry (IHC) was used to detect the corresponding protein changes in the colon tissues.

### 2.7 LC-MS/MS identification and bioinformatic analysis

Exosomal proteins were extracted using SDT buffer lysis (4% SDS, 100 mM Tris HCl, 1 mM DTT, pH7.6), and the protein concentration was determined using the BCA protein quantification kit (Bio-Rad, USA). The proteins were digested with pancreatin and processed using the ultrafiltration-assisted sample preparation (FASP) method. The peptide segments obtained after treatment were desalinated using a C18 chromatographic column, concentrated by vacuum centrifugation, and dissolved in 40 µL 0.1% formic acid.

Identification and analysis were conducted using liquid chromatography tandem mass spectrometry (LC-MS/MS). Each peptide sample was separated using a nanoliter flow rate high-performance liquid chromatography system, with mobile phase buffer A containing 0.1% formic acid aqueous solution and B containing 0.1% formic acid acetonitrile aqueous solution (84% acetonitrile). Using a 95% A-liquid equilibrium chromatographic column, each sample was loaded into a C18 chromatographic column using an automatic sampler and separated by a C18-A2 analytical column at a flow rate of 300 nL/min. After chromatographic separation, each sample was subjected to mass spectrometry analysis on a timsTOF Pro mass spectrometer. The detection method was positive ions, the ion source voltage was set to 1.5 kV, and TOF was used for detection and analysis in both mass spectrometry and tandem mass spectrometry. The scanning range of the mass spectrometry was set to 100–1700 m/z. The data parallel accumulation serial fragmentation (PASEF) mode was used to data collection. After collection, the mother ions were collected in 10 PASEF modes, with a cycle window time of 1.17 s and secondary mass spectrometry with a charge number of 0–5. The dynamic exclusion time of tandem mass spectrometry scanning was set to 24 s to avoid repeated scanning of the parent ions and generate original mass spectrometry detection data. The mass spectrometry raw data were identified and quantitatively analyzed using the LFQ (label-free quantification) algorithm in MaxQuant software (version 1.6.14).

Subcellular localization was analyzed using CELLO (http://cello.life.nctu.edu.tw/). The structural domain prediction used InterProScan software. Gene Ontology (GO) annotation passed through the GO database, and Blast2Go was used (https://www.blast2go.com/). The software annotates the GO function of all differentially expressed proteins. Cytoscape (http://www.cytoscape.org/Version 3.2.1) was used for visualization.

### 2.8 Statistical analysis

*T*-tests were used for inter-group differences, and Fisher’s exact tests were used for functional enrichment analysis. *P*-values <0.05 indicated statistically significant differences.

## 3 Results and discussion

### 3.1 Microfluidic chip fabrication and characterization

**FIGURE 1:**
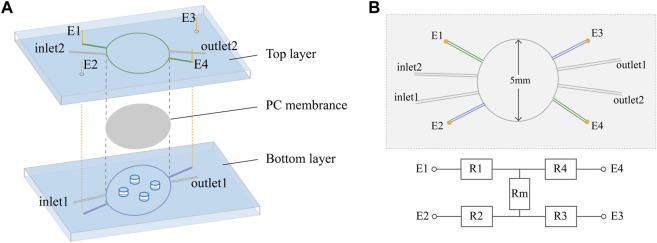
Chip design. **(A)** Exploded view of the PDMS chip showing the top layer, PC membrane, bottom layer and Ag/AgCl wire electrodes (E1, E2, E3, and E4). The diameter of the culture area on the membrane is 5 mm. **(B)** Schematic top view and simplified equivalent circuit of the chip, showing electrodes E1–E4, resistors representing the top (R1 and R4) and bottom (R2 and R3) channels, and resistor Rm representing the cellular barrier and membrane.

This work developed an in-house customized microfluidic chip that emulated the endothelial barrier microenvironment and enabled TEER monitoring. The chip was designed to comprise a semipermeable membrane that permitted the formation of an endothelial cell barrier and four electrodes to realize the calculation of resistances (Figure 1A). The circular central chamber was 5 mm in diameter and 140 μm in depth, with separate inlets and outlets for the top and bottom layers. The device consisted of three layers, including the top layer for the perfusion of medium to maintain nutrition and pressure during cell culture, the PC membrane for endothelial cellular barrier formation, and the bottom layer to mimic the extracellular matrix using hydrogel. This chip allowed easy real-time imaging and facilitated the performance of immunocytochemistry *in situ* and cell retrieval after the experiments for further analysis.

In this device, it was possible to directly measure the transendothelial or transepithelial electrical resistance (TEER), a strategy widely used for non-invasive and label-free monitoring of the tightness of the endothelial barrier *in vitro*. In the chip, four electrodes were inserted into the two channels, with two on each side of the PC membrane. In six different measurement configurations, we can directly derive isolated TEERs independent of the channel characteristics. The four electrodes were inserted into top and bottom channels some distance from the cellular barrier to avoid hampering the visual inspection of the cells, while the prestored medium in the electrode channels independent from the inlets and outlets reduced the influence of medium composition changes on the apparent TEER. From the equivalent circuit shown in Figure 1B, resistance can be determined by Gaussian elimination, including the resistance of the cellular barrier and membrane ( $R_{m}$ ). The TEER ( $\Omega {cm}^{2}$ ) is determined by the normalization of the resistance of the $R_{m}$ ( Ω ) to the culture area $A_{cult}$ ( ${cm}^{2}$ ) using Eq. 1. The TEER of the system prior to cell seeding was regarded as the baseline, which should be subtracted.

$$TEER=R_{m}∙A_{cult}=\frac{1}{4}\left(R_{1\to 2}+R_{1\to 3}+R_{2\to 4}+R_{3\to 4}-R_{1\to 4}-R_{2\to 3}\right)∙A_{cult}$$ (1)

$R_{i\to j}$ depicted the resistance between two electrodes, while *i* and *j* referred to the electrode numbers in Figure 1. The culture area on the membrane is $\pi {\left(\frac{5}{2}\right)}^{2}=6.25\pi \;\left({mm}^{2}\right)$ . This method directly measures TEER in the microfluidic chip without the need for an integrated electrode close to the cell barrier. Hence, large variations caused by non-biological sources in chips filled with culture media were eliminated.

### 3.2 Serum exosome identification

**FIGURE 2:**
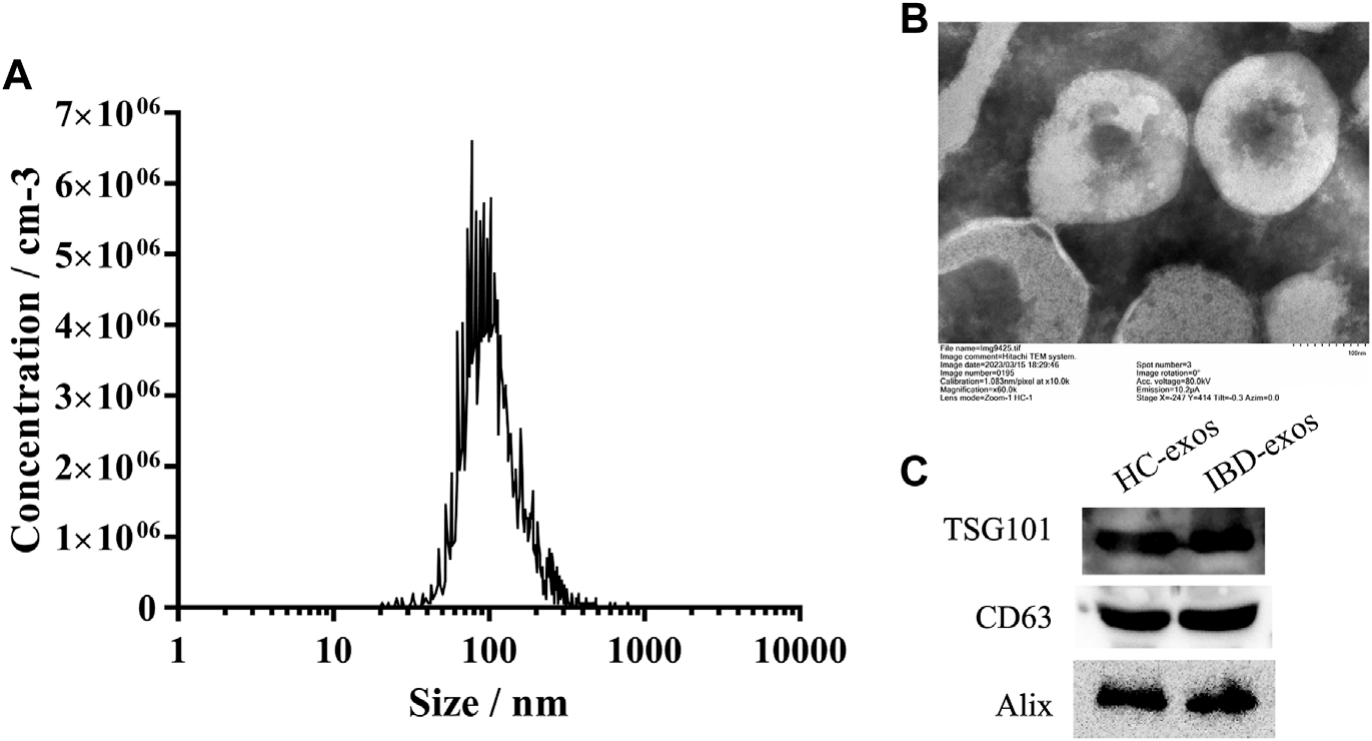
Identification of isolated exosomes. NTA result **(A)**, TEM images **(B)**, and Western blot analysis **(C)** of exosomes isolated from the serum.

The exosomes isolated from patient serum were identified by particle size analysis, image observation, and biomarker protein detection. The average particle size was 119.8 ± 63.7 nm, and 94.2% particles were distributed at approximately 101.6 nm (Figure 2A). TEM image observation showed the intact exosomes at magnification of 60 k (Figure 2B). The Western blot results demonstrated the positive detection of biomarkers for exosomes, including TSG101, CD63, and Alix proteins (Figure 2C). These data verified the characteristic of exosomes, which were then used in the subsequent experiments.

### 3.3 Exosomes from IBD serum reduce TEER

**FIGURE 3:**
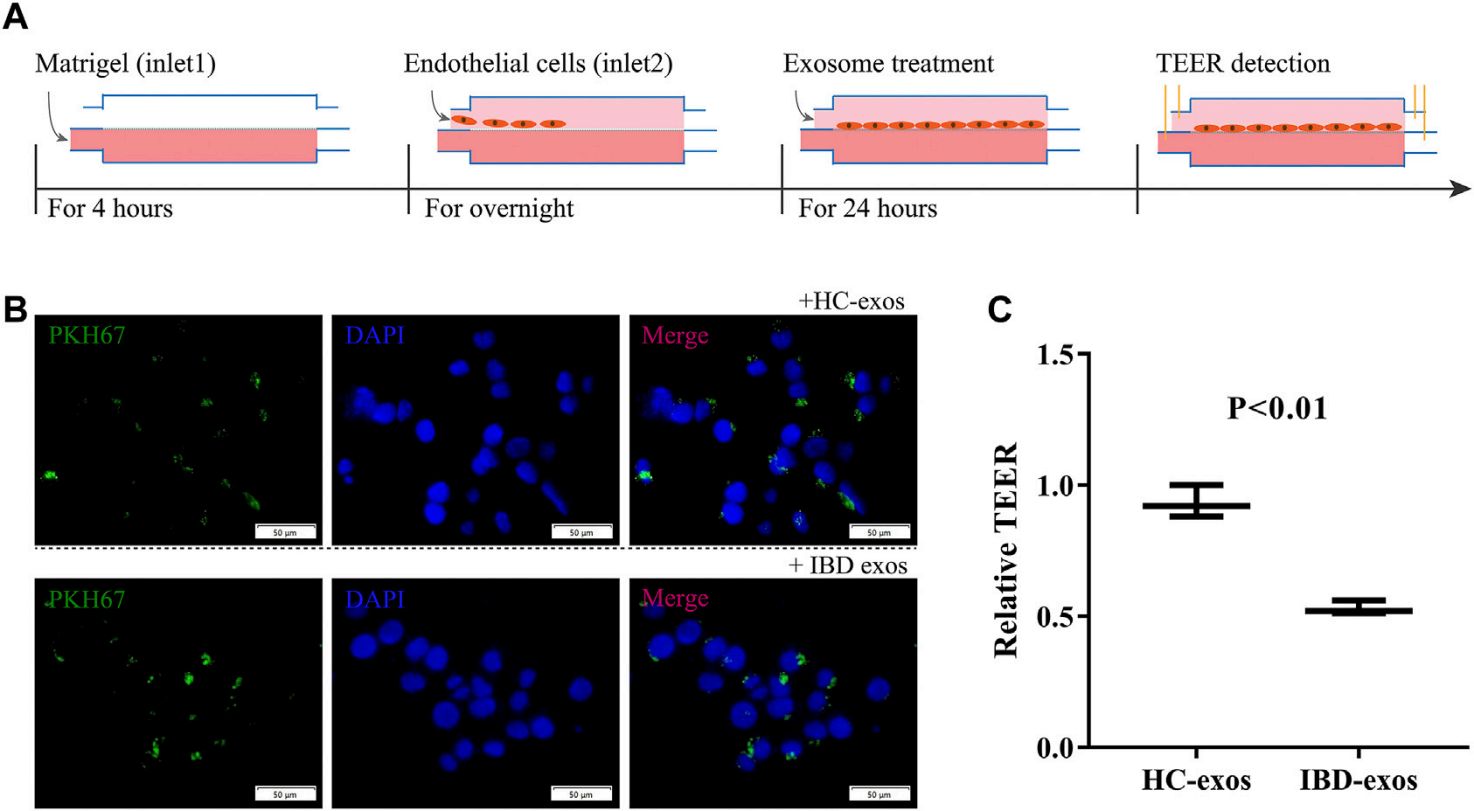
Formation of the on-chip HUVEC cellular barrier and treatment with exosomes. **(A)** Overall operation flow including the formation of the on-chip HUVEC cellular barrier, exosome treatment, and TEER detection. **(B)** Confirmation of treatment time for exosomes on the cellular barrier. **(C)** Relative TEER normalized to baseline. HC-exos, exosomes derived from healthy control serum; IBD-exos, exosomes derived from IBD serum.

A HUVEC cell line was used in this study to mimic the on-chip endothelial cellular barrier. As shown in Figure 3A, first, Matrigel was injected into the bottom chamber and incubated for 4 h for complete gelation to mimic the extracellular matrix. Then, HUVEC cells were loaded into the top chamber at a density of 10×10^6^ cells/mL and left to attach and grow on the porous membrane overnight to form the cellular barrier. The cells were maintained in a perfusion flow regime at a flow rate of 8 uL/hour using culture medium. The following day, medium containing exosomes was perfused for treatment. To confirm the treatment time, the exosomes were labeled using PKH67, a green fluorescent dye that binds to the lipid components of the membrane structure, to trace the exosomes. In the conventional culture plate, exosomes (green) were endocytosed by HUVEC cells (blue) after 24 h (Figure 3B) because the labeled exosomes were discarded if not endocytosed. The on-chip endothelial cellular barrier was then exposed to 100 μg of total exosome protein for 24 h. After that, resistances between the electrodes were measured to calculate TEER. The exosomes derived from IBD serum significantly reduced the TEER levels (*p* < 0.01), indicating that exosomes derived from IBD serum impacted the endothelial barrier (Figure 3C).

### 3.4 IBD serum exosomes promote disease progression *in vivo*

**FIGURE 4:**
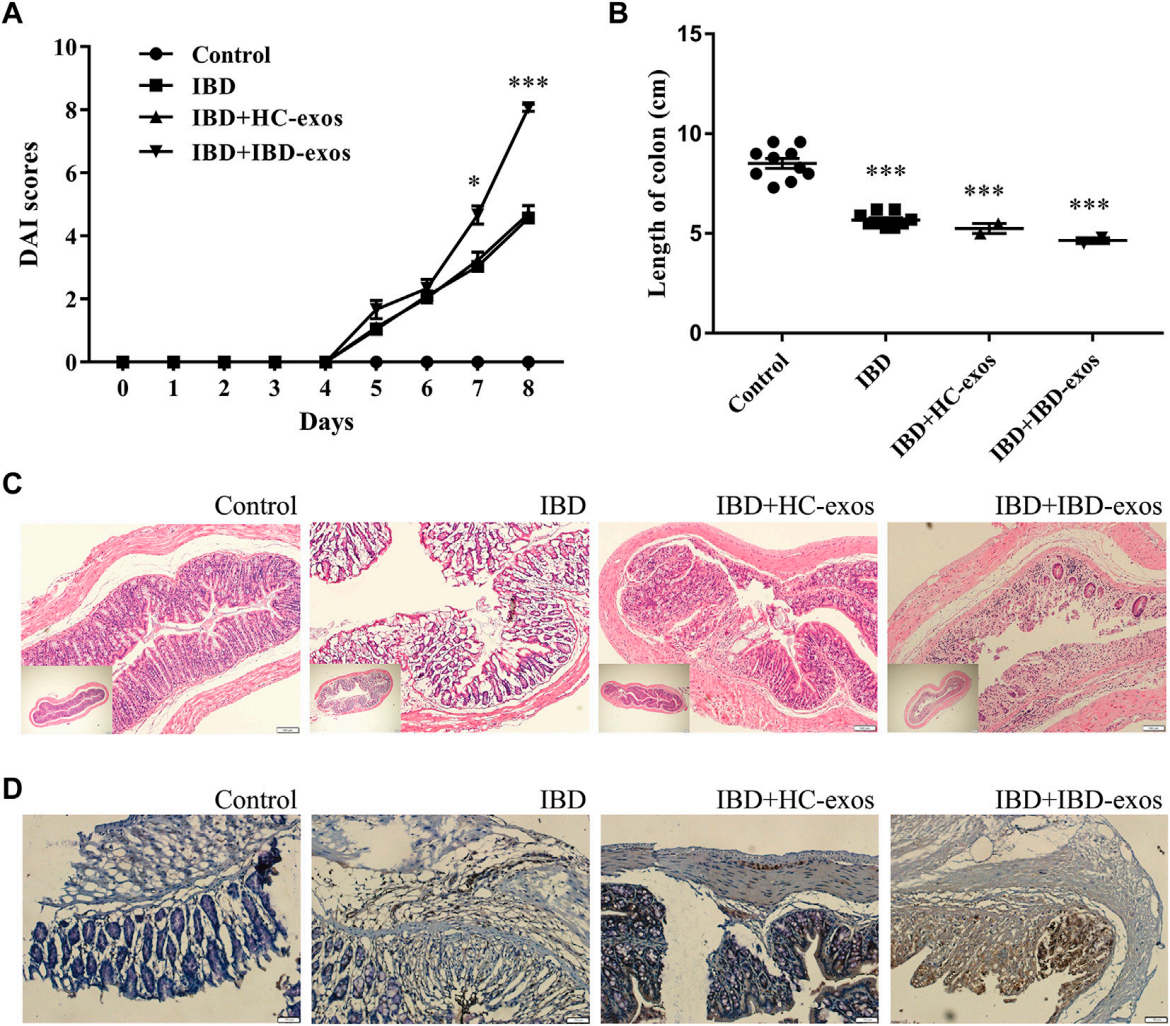
IBD serum exosomes promote disease progress in the mouse model. DAI scores **(A)**, length of colons **(B)**, HE staining **(C)**, and IHC detection of S100A8 protein **(D)**.

To validate the effect of serum exosomes on the progression of IBD *in vivo*, a mouse IBD model induced by DSS was established. Exosomes derived from control or IBD patients were injected through the tail vein every other day from the third day after DSS treatment. Mice in the IBD group, regardless of exosome treatment or not, showed higher DAI scores than those in the normal control group at day 5, indicating the effects of DSS induction. To day 7, mice in the IBD group treated with exosomes derived from IBD patients showed significantly higher DAI scores than all the other three groups. The levels continue to increase until day 8, and the mice showed much more severe illness (Figure 4A). All the mice were euthanized on day 8, and the lengths of colons in the IBD group treated with IBD exosomes were significantly shorter than those of the control group (Figure 4B). HE staining also showed that the IBD group treated with IBD exosomes had the most serious disease (Figure 4C). Additionally, S100A8, which plays an important role in inflammation and angiogenesis (Li et al., 2012), detected by IHC showed greatly enhanced expression in the colon tissues of the IBD exosome-treated group (Figure 4D). These data supported that exosomes derived from IBD patients significantly promoted disease progression.

### 3.5 Proteomic profiling of the exosomal protein cargoes

**FIGURE 5:**
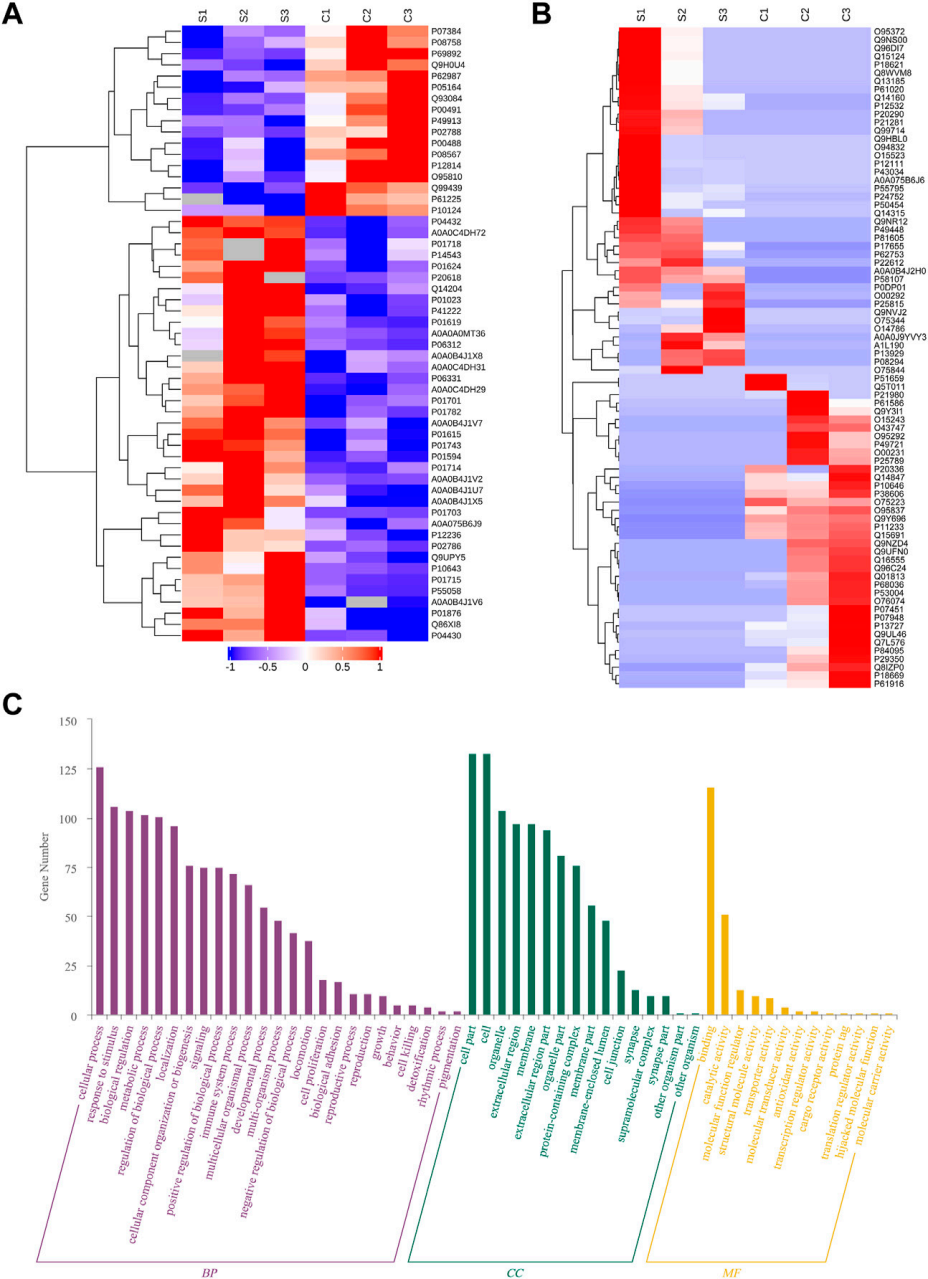
Profiling of IBD serum exosomes. Heat map of differentially expressed proteins **(A)** and proteins only present in IBD or control serum exosomes **(B)**. Biological process (BP), cellular component (CC), and molecular function (MF) analysis **(C)**.

Although the impact of serum exosomes on IBD progress was validated, the corresponding regulatory protein cargoes were still not clear. Therefore, we further profiled the differential proteome of IBD serum exosomes. In total, 5765 peptides and 891 proteins were identified, including transthyroxine (TTR) and *ß*-2-glycoprotein 1 (APOH), which were consistent with the common proteins in serum exosomes listed in the ExoCarta (http://www.exocarta.org) public database. Quantitative analysis screened a total of 55 differential (38 upregulated and 17 downregulated) proteins in IBD serum exosomes compared with control (Figure 5A). Meanwhile, 38 proteins were screened as only present in control exosomes (not identified in IBD), and 42 proteins were screened as only present in IBD exosomes (not identified in control) (Figure 5B). Among these identified differential proteins, many immunoglobulins were significantly upregulated, showing that immune factors, especially abnormally expressed immunoglobulins, contributed to IBD pathogenesis. Transferrin receptor protein 1 (TFRC) was significantly upregulated by more than five-fold in IBD exosomes. Transferrin receptor levels are reportedly increased in inflammatory tissues, while HIF *a* pathway agonists may benefit such patients by increasing non inflammatory tissue-mediated iron absorption and reducing mucositis (Fagundes et al., 2022). Annexin A5 (ANXA5), a calcium and phospholipid-binding protein, was significantly downregulated in IBD exosomes. ANXA5 reportedly has potential inhibitory effects on inflammation through binding to aged red blood cells, activated platelets, endothelial microparticles, and tumor blood vessels (Domeij et al., 2013), effectively alleviating TNBS-induced colitis by inhibiting inflammatory cell infiltration (Zhang et al., 2021), which may be a potential target in the treatment of IBD.

Analyses of biological processes, cellular components, and molecular functions were also conducted on the screened differential proteins. As shown in Figure 5C, these differential proteins were mainly enriched in biological processes such as intracellular processes, stimulus responses, biological regulation, metabolic processes, intracellular component biosynthesis, and signaling; the cell components included organelles, extracellular areas, and cell membranes; and the molecular functions involved binding, catalytic activity, molecular function regulation, structural molecular activity, transporter activity, molecular transduction activity, antioxidant activity, and transcriptional regulation.

## 4 Conclusion

This study presents an on-chip endothelial cellular barrier integrated with electrodes to detect TEER changes after treatment with exosomes derived from IBD serum. The IBD serum exosome significantly reduced TEER values. In the *in vivo* mouse models, the group exposed to exosomes from IBD serum showed higher DAI scores, shorter colons, more severe histological features, and higher levels of S100A8 expression, which promoted disease progression. The exosomal protein cargoes were further explored using proteome profiling, in which TFRC and ANXA5 showed great potential as novel regulators in IBD.

## Data Availability

The datasets presented in this study can be found in online repositories. The names of the repository/repositories and accession number(s) can be found at: www.proteomexchange.org, PXD043594 and www.iprox.cn/page/project.html?id=IPX0006559000.
